# Signatures of native-like glycosylation in RNA replicon-derived HIV-1 immunogens

**DOI:** 10.1039/d5cb00165j

**Published:** 2026-01-12

**Authors:** Himanshi Chawla, Jacob T. Willcox, Grace M. Hayes, Murillo Silva, Wen-Hsin Lee, Gabriel Ozorowski, John Butler, Paul F. Mckay, Robin J. Shattock, Andrew B. Ward, Darrell J. Irvine, Max Crispin

**Affiliations:** a School of Biological Sciences, University of Southampton Southampton SO17 1BJ UK max.crispin@soton.ac.uk; b Koch Institute for Integrative Cancer Research, Massachusetts Institute of Technology Cambridge MA USA; c Department of Integrative Structural and Computational Biology, The Scripps Research Institute La Jolla CA 92037 USA; d Center for HIV/AIDS Vaccine Development, IAVI Neutralizing Antibody Center and the Collaboration for AIDS Vaccine Discovery (CAVD), The Scripps Research Institute La Jolla CA 92037 USA; e Department of Infectious Diseases, Imperial College London Norfolk Place London W2 1PG UK

## Abstract

RNA-based vaccines have emerged as a highly effective delivery platform. However, this approach eliminates the possibility for immunogen purification, common in manufacturing of recombinant immunogens. In HIV-1 vaccine design, this is of particular importance because non-native epitopes can compromise the desired immune response, and native immunogen assembly is important for presentation of glycan-based epitopes targeted by broadly neutralizing antibodies. Here, we investigate the assembly and glycosylation of the archetypal trimeric HIV-1 immunogen, BG505, in the soluble single-chain format (NFL.664) that bypasses the need of maturation by furin cleavage. We have investigated the presence of the trimer-associated mannose-patch as oligomannose-type structures at these *N*-linked glycosylation sites are indicative of native-like glycoprotein structure. Despite the presence of features of native-like glycosylation, both electron microscopy and glycopeptide analysis indicated the presence of a sub-population of non-native material. We also investigated the glycosylation of material derived from cell-types that likely produce immunogens near the site of intramuscular RNA injection. We show that replicon-transformed dendritic and muscle cell lines generate immunogens displaying similar oligomannose-type glycan content, whereas sites presenting complex-type glycosylation differed substantially in the levels of glycan processing. Overall, the control of the immunogen assembly by protein engineering is sufficient to drive native-like glycosylation at the majority of glycosylation sites independent of producer cells. Furthermore, we explored the engineering of RNA immunogens to improve glycan site occupancy. Controlling immunogen assembly at the nucleotide level offers a route to enhanced RNA-based immunogens.

## Abstract 2

Assembly and glycosylation of an HIV-1 immunogen from an RNA replicon are described. Ntive-like glycan processing and assembly predominate, with cell-specific glycosylation in muscle and dendritic cells. Nucleotide editing improves glycan occupancy.

## Graphical abstract

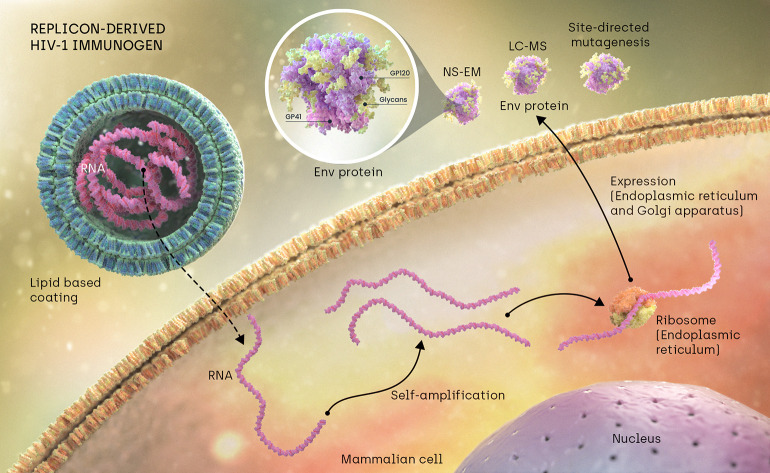

## Introduction

The response to the recent pandemic has highlighted the value of adenoviral^1^ and RNA-based immunogens.^2^ A major advantage is the speed at which they can be deployed in comparison to traditional protein-based immunogens.^3–5^ However, one potential limitation is that the viral immunogens must be entirely encoded by the nucleotide sequence and there is no opportunity for post-expression purification that is typically implemented in the production of recombinant-based immunogens.^6–8^ Therefore, there is considerable interest in understanding how RNA-based and adenoviral-based immunogens are assembled *in situ*^9,10^ and how nucleotide editing can help shape this process.^11^ In HIV-1 vaccine research, understanding immunogen assembly is particularly important because it is thought that in order to elicit a broadly neutralizing antibody (bnAb) response^12^ the immunogen must display native-like assembly,^13–17^ post-translational modifications,^18–20^ and minimal amounts of non-native material.^17,21–24^ One of the leading HIV vaccine strategies aiming at eliciting bnAbs does however entail targeting particular B cell lineages with highly engineered immunogens but this strategy also includes immunization with a final ‘polishing immunogen displaying native-like features’.^25–28^

The HIV Envelope glycoprotein (Env) has emerged as the main focus for HIV-1 vaccine design and there have been considerable developments in the production of native-like Env glycoproteins.^24,29–35^ Manufacturing of the HIV-1 viral spike has proved challenging as the viral glycoprotein is metastable and requires complex post-translational maturation. In a native setting, the *env* gene is translated as a gp160 polypeptide which is heavily glycosylated and is proteolytically cleaved *via* furin into gp120 and gp41 heterodimers which trimerize to form a spike on the virus surface.^36,37^ A breakthrough strategy in recombinant production involved solubilizing the protein by adding a stop codon at position 664 to prevent the translation of the transmembrane domain, disruption of the fusion mechanism of gp41 by the insertion of I559P mutation, and the use of stabilizing disulfide bonds, collectively referred to as SOSIP.664.^15,29,33^ This format is dependent on furin cleavage, and despite optimization of the furin cleavage site, can still exhibit incomplete cleavage and the resulting material usually requires post-expression purification.^7^

The dependence on protease cleavage can be bypassed using single-chain constructs, such as the natively flexibly linked format (NFL.664).^38–40^ The assembly and glycosylation of NFL.664 and SOSIP.664 are known to be highly similar and are of particular interest in nucleotide-based immunogen design.^40^ However, as these recombinant formats generally require post-production purification, we are interested in the assembly of RNA-derived immunogens where this process is precluded and where there is consequently an elevated potential for the production of non-native-like material. Glycan processing is highly sensitive to three-dimensional macromolecular architecture^41,42^ and we can utilize site-specific analysis of potential *N*-linked glycosylation sites (PNGS) to monitor immunogen assembly.^43,44^

One such important signature in immunogen assembly is the preservation of under processed oligomannose-type glycan structures. Previous studies have demonstrated the presence of an extended cluster of oligomannose-type glycans, known as the intrinsic mannose patch (IMP), on the outer domain of gp120.^45^ The IMP sites are known to be highly conserved^46^ and a target of numerous broadly neutralizing antibodies.^12^ This region is a characteristic of monomeric gp120 and previous analyses of recombinant native-like trimers and virion-derived Env have demonstrated the presence of an additional ‘trimer-associated mannose patch’ (TAMP) on the native-like trimer.^42,47^ The presence of these glycans at protomer interfaces is linked to the quaternary structure of the protein and they usually arise due to steric occlusion of glycan processing enzymes.^48^ These glycans often contribute to the epitopes of many broadly neutralizing antibodies and can also influence immunogen trafficking.^49,50^ In contrast to oligomannose-type glycans, which are predominantly dependent on the macromolecular architecture, the processing of complex-type glycans is known to be substantially influenced by the host cell.^51,52^ Fine glycan processing in complex-type glycans, such as changes in sialylation or fucosylation can also influence antibody binding.^53,54^

In this study, we utilize glycopeptide analysis to investigate whether RNA- and protein-based immunogens exhibit the same protein-dependent glycan synthesis. We examined the production of immunogens arising from the alphavirus-based replicon system.^55^ This system exhibits RNA self-amplification within the target cell *via* the transcription of non-structural proteins which copies the replicon RNA in addition to desired proteins, in this case BG505 NFL.664 Env. Although RNA replicons are an effective platform for delivering viral glycoprotein,^56–59^ the requirement for high-fidelity folding in HIV-1 has prompted us to examine the assembly and processing of the Env. We further investigate how assembly can be influenced by nucleotide editing.

## Results and discussion

### Expression and characterization of single-chain replicon HIV-1 immunogen

To investigate the entire population of Env products produced by replicon-mediated expression we analyzed material captured by a broad-acting lectin. The resulting Env was analyzed by negative stain-electron micrography (NS-EM) and liquid chromatography–mass spectrophotometry (LC-MS).

**Fig. 1:**
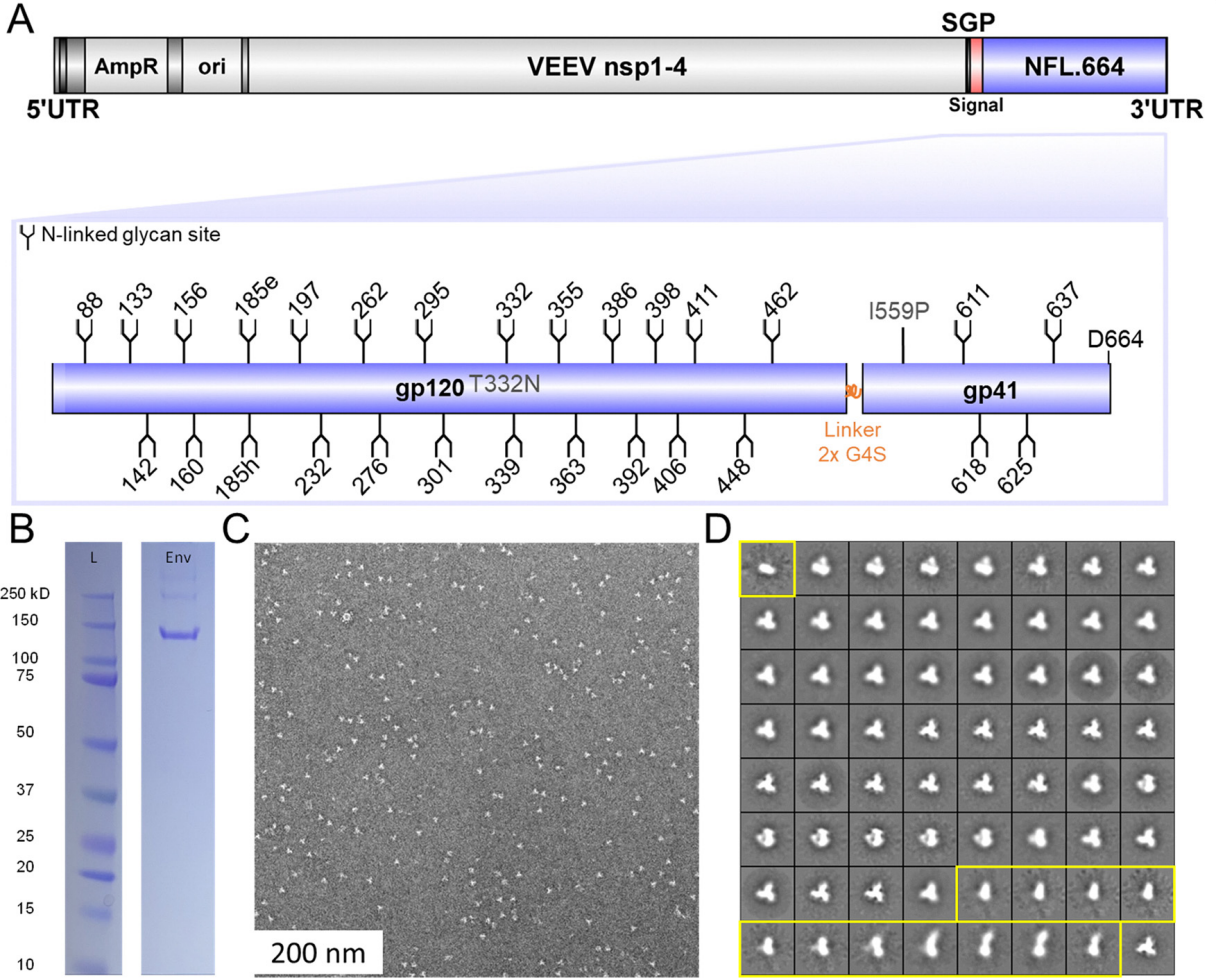
Expression of replicons encoding soluble single-chain HIV-1 immunogen (BG505 NFL.664) (A) schematic representation of the BG505 NFL.664 replicon constructs. The domains of the construct illustrated are: Venezuelan equine encephalitis vector (VEEV), non-structural proteins (nsp's), sub genomic promoter (SGP), ampicillin resistance (AmpR), origin of replication (ori), untranslated region (UTR). (B) SDS-polyacrylamide gel electrophoresis analysis of the replicon expressed BG505 NFL.664 Env in human embryonic kidney (HEK) 293F cells purified using *Galanthus nivalis* (GNL) lectin beads. (C) Representative of NS-EM image of BG505 NFL.664 Env particles secreted by HEK 293F cells transfected with replicon RNA. (D) 2D class averages of the particles. Amongst all of the particles imaged, 17% fall within class averages corresponding to non-native conformations shown in yellow.

We utilized a replicon system derived from the Venezuelan equine encephalitis virus (VEEV) for the expression of HIV-1 Env. The VEEV genome encodes for non-structural proteins (nsp1–4) including the replicase (RNA-dependent RNA polymerase complex) and the structural genes, separated by sub genomic promotor (SGP), which drives the transcription of structural proteins of the RNA, thus has a potential for sustained expression. We have used the VEEV genome as a backbone as described previously^58^ with structural genes replaced with a single-chain natively flexible linked HIV-1 Env trimer of the clade A strain of HIV-1, BG505. The design of the so-called BG505 NFL.664 trimer has been described previously^39,40^ and is summarized in Fig. 1A.

To express Env using the VEEV replicon, we first transcribed the plasmid DNA containing the VEEV's genome and BG505 NFL.664 genes into RNA using an *in vitro* transcription (IVT) reaction.^57,58^ The transfection of purified RNA in human embryonic kidney (HEK) 293F cells was carried out using neon-electroporation for initial optimization and TransIT-mRNA to bulk up the protein expression for LC-MS analysis. After 72 h of transfection, the supernatant was further purified using *Galanthus nivalis* lectin (GNL). We selected GNL as it binds to the core of all *N*-linked glycans and thereby ostensibly facilitates the analysis of the whole Env fraction, regardless of its native configuration. As replicon-based immunogen delivery eliminates post-expression purification, we utilized GNL beads to purify both the natively and non-natively folded Env material. We analyzed the protein by SDS-PAGE (Fig. 1B). Further, to determine the proportion of Env displaying native-like conformation, we performed NS-EM analysis of the replicon-expressed protein. Using NS-EM, we established that 83% of the total protein structure is trimeric and 17% constitutes non-native material (Fig. 1C and D). Non-native material has been shown to elicit non-neutralizing antibody responses.^21,22^

Furthermore, to assess that the broad specificity lectin is not capturing endogenous *N*-linked glycoproteins, we performed NS-EM analysis of Env purified with GNL followed by size exclusion chromatography (Fig. S1) and Env purified with VRC01 (Fig. S2). Both antibody-based purification and GNL-based purification exhibited comparable levels of non-native conformations, suggesting that non-native conformations are mostly specific to Env. However, there remains possibility of minor elution of endogenous secreted *N*-linked glycoproteins in both purification methods.

### Site-specific glycan characterization of replicon expressed HIV-1 Env protein

**Fig. 2:**
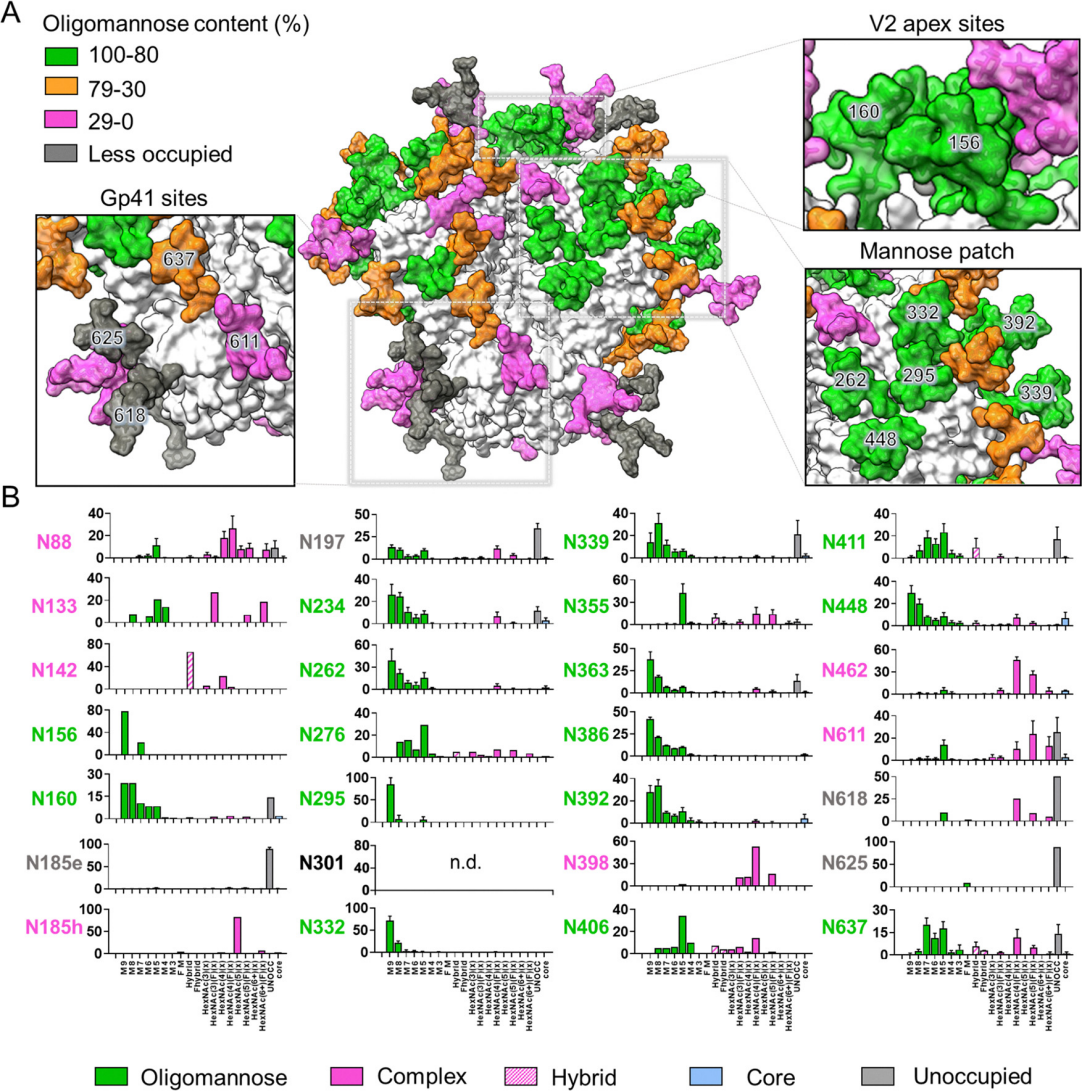
Glycosylation of the replicon expressed soluble NFL Env trimer. (A) Representative glycans are modelled onto the HIV-1 Env trimer (PDB ID 5C7K).^68^ All the 28 *N*-linked glycans are mapped on the model based on abundance of oligomannose content as defined by the key. Less or unoccupied sites are highlighted in gray. (B) Site-specific analysis of the replicon expressed BG505 NFL.664. Three or more biological replicates of BG505 NFL.664 were produced in HEK293F to generate the glycan compositions. Oligomannose-type glycans Man_3_GlcNAc_2_ to Man_9_GlcNAc_2_ are represented as M3 to M9 and fucosylated glycans are represented as F. Bars represent the mean ± the standard error of the mean of the biological replicates of expressed protein analyzed. The bar graphs without error bars show data from fewer than three biological replicates.

We utilized LC-MS analysis of glycopeptides derived from the whole Env pool, defined by capture by a broad-acting lectin, to characterize the glycosylation at the site-specific level. We sought to resolve the site-specific glycan compositions of three or more biological replicates of replicon expressed Env protein in mammalian cells, HEK 293F (Fig. 2A and B). To quantify the glycan compositions at each site, we digested glycopeptides using three protease enzymes, trypsin, chymotrypsin, and alpha lytic protease (ALP). These proteases were utilized to generate glycopeptides containing a single *N*-linked glycan sequon and the resulting glycopeptides were analyzed using LC-MS. We were able to determine the glycan compositions across the 28 *N*-glycan sites, however there were a few sites for which we could not obtain replicates, thus these are displayed without error bars (Fig. 2B). To display the site-specific glycan processing state in their 3D context, we modelled the glycan composition onto the trimeric Env based on the percentage of oligomannose-type glycan present at each site (oligomannose-type, 100–80%; mixed, 79–30%; and complex-type glycans, 29–0%; Fig. 2A).

Prior studies demonstrated that changes in the abundance of under-processed glycan structures can indicate changes in the fine quarternary structure of the protein.^60,61^ To determine the presence of native-like glycan signatures, we compare the glycosylation patterns across RNA replicon expressed Env in this study (Fig. 2A, B and Fig. S4) with previously analyzed virion-derived Env^52,62^ and recombinant Env.^40,48,63,64^ Our analysis revealed a highly similar oligomannose-type and hybrid-type glycan content, with localized variations occurring. We observed a high proportion of oligomannose-type glycans within the IMP and TAMP, consistent with native-like architecture.^42,48^ For example, N332 within IMP displayed 100% oligomannose-type glycans with a predominance of Man_8–9_GlcNAc_2_ glycan structures (Fig. 2A and B).

In contrast to oligomannose-type glycans, complex-type glycans are typically not significantly influenced by the protein structure of Env but rather by the host cell used for expression.^51^ We observed complex-type glycans at several sites including N88, N133, N142, N185h, N301, N398, N462 and the gp41 sites N611 and N618 (Fig. 2A and B). This is consistent with the glycan composition observed in recombinant production *via* plasmid DNA in this study (Fig. S4) and with previous observations of native viral spike and recombinant Env protein.^40,52,63,64^

Another parameter which influences the elicitation of antibodies is glycan site occupancy, as so-called ‘glycan holes’ have the potential to elicit non-neutralizing antibodies that can dilute the desired immune response.^65^ In our analysis, we noted several sites on replicon expressed Env showing a population of unglycosylated peptides containing PNGS, N185e, N197 and within gp41 at sites, N611, N618, and N625 (Fig. 2A and B). Similar underoccupancy has been observed previously within recombinant native-like trimers and has resulted in non-neutralizing antibody responses.^11,52,66,67^ For example, the RM20E1 antibody isolated from a BG505 SOSIP.664-immunized macaque binds to the glycan hole at N611.^11,65^

Overall, our glycosylation analysis reveals the glycan signature characteristic of a native-like trimer on Env derived from replicon RNA. As an increasing number of RNA-based vaccine candidates are being developed, glycan analysis offers a route to probe immunogen integrity and to give insight into the scope for improving immunogen assembly.

### Non-native material exhibits near native-like glycosylation

While the majority of the glycosylation sites within the intrinsic mannose patch display under-processed oligomannose-type glycans, any changes in glycan processing at these sites can provide an insight about structural changes in the trimeric structure of Env. Thus, glycans at these sites can be used as a sensitive reporter to signify the presence of non-native constituents in the expression. From our NS-EM data, we revealed that a small proportion of the Env material expressed by replicon RNA contains non-native material. To confirm whether the presence of non-native material impacts immunogen glycosylation, we compared the site-specific glycan analysis of replicon-expressed Env purified *via* GNL- and PGT145-affinity chromatography, and the material not retained by the PGT145 affinity column. The latter material would likely contain non-trimeric Env that could be present in the GNL-purified material.^7^ To detect the differences in glycan processing, we can first consider the glycan processing of oligomannose-type glycans at the IMP as these sites are under less influence from quaternary structure and their glycan processing states are known to be indicative of native-like conformations within the gp120 outer domain.^45,48^ We could not obtain glycosylation composition of all the TAMP sites of Env produced by PGT145 purification. However, in the GNL purified material we observed native-like glycosylation at the TAMP sites demonstrating native-like glycan signatures independent of a quaternary architecture-specific purification stage (Fig. 2).

**Fig. 3:**
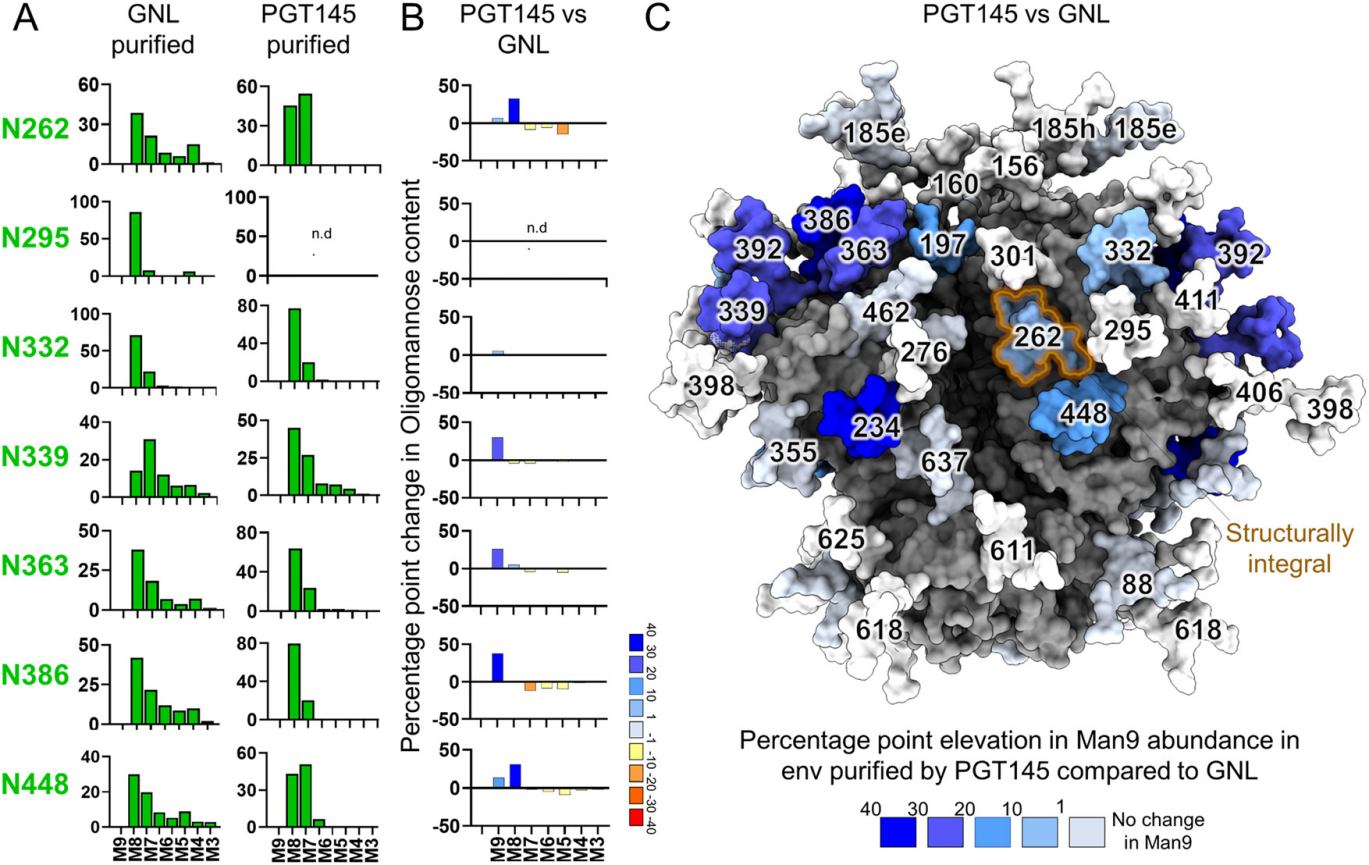
Comparison of the glycan composition of replicon expressed BG505 NFL.664 Env purified using GNL and PGT145 affinity purification. (A) Representation of glycan composition of the intrinsic mannose patch sites on Env purified *via* GNL, PGT145, and flow through of PGT145 purified by GNL. Oligomannose-type glycans Man_3_GlcNAc_2_ to Man_9_GlcNAc_2_ are represented as M3 to M9. (B) Percentage point difference in oligomannose-type glycans between PGT145- and GNL-affinity purified material. (C) A map of the differences in Man_9_GlcNAc_2_ (Man9) abundance between Env purified by PGT145 and GNL affinity purification plotted on a model based on PDB 5C7K. The structurally integral glycan site, N262 is shown with orange outline. The sites which were not determined are shown in white; n.d., not determined. The bar graphs (GNL purified data) represent the mean quantities of three or more biological replicates, with error bars representing the standard error of the mean. The bar graphs without error bars show data from fewer than three biological replicates.

The glycan at N262 is highly conserved as the protein-proximal stem of the glycan is largely buried in a protein cleft.^69–71^ Due to steric hindrance, this site is exclusively dominated by oligomannose-type glycans on HIV-1 Env and presents predominantly Man_9_GlcNAc_2_ glycan structures.^40,42,52,64^ We observed similar glycan compositions on Env purified by GNL and PGT145, however, our GNL-purified Env and Env present in flow through of PGT145 shows a small population of glycans showing elevated trimming, Man_6–8_GlcNAc_2_ (Fig. 3A). This is consistent with the presence of a non-native fraction in the GNL-purified Env which is eliminated when we purified with a trimer-specific antibody. The N332 site presents less processed oligomannose-type glycans in both GNL and PGT145 purified Env which is consistent with previous findings.^40,63^ We observed higher glycan processing at other IMP sites, N339, N363, N386, and N448 on both GNL purified Env and Env present in flow through of PGT145 (Fig. 3A). The Env material present in the flow through of PGT145 presents enhanced glycan processing at N448 and underoccupancy at N339 and N363. In addition to glycan processing, we investigated the binding properties of Env purified with GNL, and Env from the PGT145 flow-through, with 2G12 and PGT121 bnAbs, and the non-nAb, F240. Both Env samples showed binding to the glycan-dependent antibody, 2G12 and anti-V3 antibody, PGT121 (Fig. S3). Env present in the flow through of the PGT145 affinity column bound with the non-nAb F240, whereas the GNL-purified Env showed negligible binding with the F240. This suggests the presence of high proportion of non-native material in the flow through of the PGT145 affinity purification. However, the low expression levels of Env from RNA-based immunogens means that the antibody panel described here is limited in providing a comprehensive view of the binding properties.

To explore the glycosylation changes across all *N*-glycan sites on Env protein, we plotted percentage point differences in Man_9_GlcNAc_2_ presented by Env purified *via* PGT145 and GNL (Fig. 3B). We observed a predominance of Man_9_GlcNAc_2_ glycan content at several sites outside of the IMP, such as at N197, N234, N392 on Env purified by PGT145 compared to GNL (Fig. 3B). This suggests that the total material in GNL-purified Env does contain some constituents which allow the material to be more processed and thus reflect changes in structural conformation consistent with the NS-EM observations. These non-native constituents are eliminated when we purify the protein with PGT145 which binds only to Env which presents a native-like quaternary trimeric structure. Overall, our findings suggest that the elimination of post-production purification in replicon-expressed Env exhibits some proportion of Env displaying slightly enhanced glycan processing. This reflects the presence of some non-natively folded constituents which is consistent with the NS-EM results.

### Cell-directed glycosylation changes in Env protein expressed *via* replicon

**Fig. 4:**
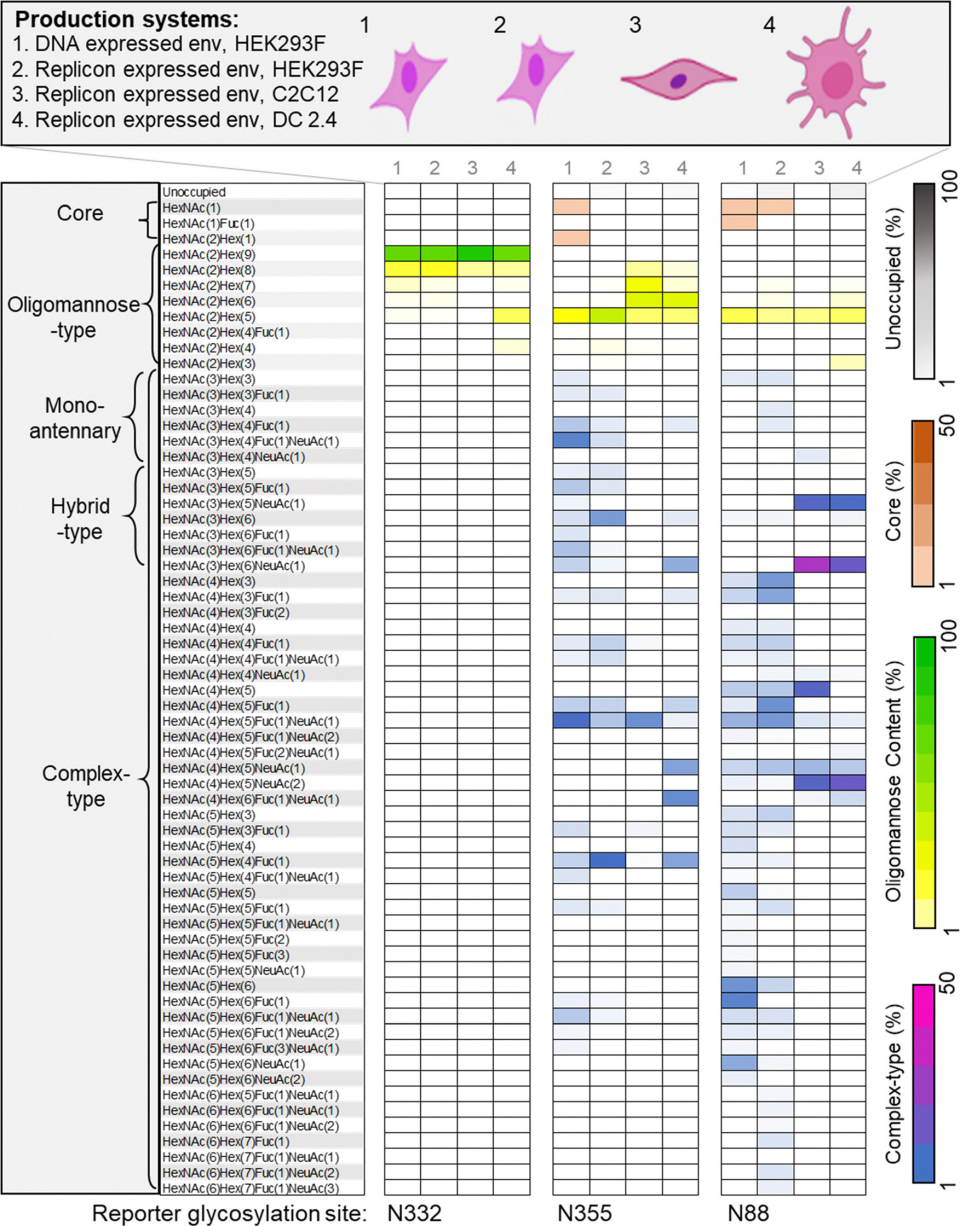
Differential glycan composition on Env expressed in different cell lines. Glycan composition at reporter glycosylation sites predominantly displaying: oligomannose-type glycans (N332), a mixture of complex- and oligomannose-type glycans (N355), and complex-type glycans (N88). Four different production systems were used as described in the key, including expression in three different cell lines, HEK293F, C2C12 and DC2.4 cells. All the glycan composition observed in the site-specific analysis are shown on the left, simplified in distinct categories represented as, core, oligomannose-type, and complex-type glycans. Some compositions annotated as complex-type glycans can exhibit isomers formally constituting hybrid-type glycans. Represented glycan compositions (See Methods for full classification) are colored according to the scale provided in the right for each category of glycan composition. The representative glycan composition data at all the sites is the average of three or more biological replicates.

We next analyzed the glycan composition of the replicon expressed Env in different cell lines, including mouse muscle (C2C12) and dendritic (DC2.4) cell lines. We chose these cell lines as models of those cell types potentially predominant at the site of intramuscular RNA injection. To understand the glycan composition changes, we produced Env using four different production systems as described in Fig. 4. We performed three biological replicates to generate the site-specific analysis data across different cell lines, including HEK 293F, DC2.4, and C2C12 (Tables S1–S3). We observed similar oligomannose-type glycans from across these cell lines, with conserved IMP and TAMP glycan processing. These observations are consistent with the previous finding that HIV-1 Env glycosylation is heavily influenced by the quaternary structure of the protein as glycan processing is conserved at many sites despite changes in the cell line used for expression or culture conditions.^45^ However, we detected divergent glycan processing of hybrid- and complex-type glycans on replicon expressed Env in different cell lines. For example, Env produced in C2C12 and DC2.4 cells presented elevated levels of processing of complex-type glycans compared to those from Env expressed in HEK 293F cells (Fig. S4). We observed high glycan occupancy and processing at gp41 sites on Env expressed in C2C12 cells. Whilst there are differences in glycosylation at a site-specific level between Env derived from different cell lines, there were no substantial differences in Env glycan compositions when comparing material, from the same cell-type, when expressed from plasmid DNA or replicon RNA (HEK 293F; Table S4 and Fig. S4). This highlights that Env glycan composition is largely independent of the immunogen delivery platform investigated in this study.

To highlight the differences in site-specific glycan processing, we selected one site that presents a high abundance of under-processed oligomannose-type glycans (N332), one that presents both complex- and oligomannose-type glycans, referred to as a mixed site (N355), and one that presents predominantly complex-type glycans (N88). At the N332 glycosylation site, we observed a predominance of less processed Man_8–9_GlcNAc_2_ glycan compositions in Env derived from all four expression systems. However, we observed elevated levels of the more processed Man_5_GlcNAc_2_ glycan on dendritic cell-derived Env, but this glycan constituted only ∼10% of the population and Man_8–9_GlcNAc_2_ remained the most abundant glycan species.

The N355 and N88 glycosylation sites presented a diverse mixture of oligomannose-, hybrid-, and complex-type glycans across all expression systems. However, N355 showed a higher abundance of oligomannose-type glycans compared to N88, which presents 80–90% complex-type glycans (Fig. 4). The population of oligomannose-type glycans observed at both these sites are more processed than those of N332, consistent with lower steric exclusion. Furthermore, the population of hybrid- and complex-type glycans observed at these sites are quite diverse. Compared to N355, the N88 site showed greater amounts of hybrid-type glycans on Env derived from muscle and dendritic cell lines (Fig. 4). The presence of hybrid-type glycans is noteworthy as they can form targets for bnAbs.^72^

In contrast to oligomannose-type glycans, the attachment of different monosaccharides within complex-type glycans is more heavily influenced by the host cell,^73^ culture conditions or supplements provided for protein expression.^74^ For example, in contrast to Env derived from C2C12 and DC2.4 cells, material produced in HEK 293F cells displayed a high percentage of fucosylated species (∼40%) and a lower percentage of sialylated species (10–20%) (Fig. S5). These observations are consistent with previous studies of glycoprotein derived from HEK 293F cells which have shown high levels of fucosylation but low sialylation.^75,76^ Similarly, the glycan compositions in mouse serum have found to be more sialylated than in human serum, while fucosylation was more common in human serum than in mouse serum.^77,78^ In this study, we observed similar finding in case of murine muscle and dendritic cells expressed protein revealed high sialylated proteins compared to the protein expressed in human embryonic kidney cells (Fig. S5 and Fig. 4).

Overall, these finding are consistent with earlier observations that cell origin can impact Env glycosylation.^51,79,80^ These features of glycan heterogeneity are important to note as they can influence the function of proteins, such as in immune modulation.^73,81^ Whilst the glycosylation of complex and mixed sites on Env is heavily influenced by producer cells, the occupancy at all glycan sites tend to be more conserved, although we do detect some variations (Fig. 4 and Fig. S4). The differential occupancy of highlighted sites in Env, such as being low in mouse dendritic cells but high in muscle cells, may potentially result in skewed immunodominant responses.^82,83^ Glycan heterogeneity is one of the contributing factors to the immunodominance of many of the non-neutralizing and autologous neutralizing epitopes.^84–86^ This mixed glycan occupancy could result in a complex interplay of immune responses, potentially affecting the efficacy of vaccines or therapeutic strategies targeting specific epitopes. However, these findings underscore the influence of the producer cells on Env glycosylation, it will be important to extend these observations into *in vivo* settings.^49,53,72^

### Targeted repair of glycan occupancy

RNA-based immunogens eliminate the possibility of post-expression purification of the desired product and thus improvements in immunogen assembly can only be achieved at the nucleotide level. In this study, we observed various features of replicon expressed Env glycosylation, which could elicit non-neutralizing antibodies. One of the features is glycan underoccupancy which is an important parameter to consider in the case of HIV-1 immunogen design. In our site-specific analysis data, we observed *N*-glycan sites which present low occupancy, including N185e, N197, N611, N618, and N625 (Fig. 2A and B). To improve the glycan occupancy, we utilized an approach previously applied by Derking *et al.*^11^ on recombinant HIV-1 Env to improve the glycan occupancy. The rationale behind this strategy is that the enzyme involved in glycan attachment from the dolichol phosphate to the asparagine site, oligosaccharyltransferase (OST)^87,88^ has a higher affinity towards NXT than NXS glycan sequons.^11,89^ We sought to apply the same strategy to replicon expressed Env and chose three sites presenting low occupancy and having serine at the third position of the *N*-linked glycosylation sequon N185e, N197 and N611 for targeted repair of glycan occupancy.

**Fig. 5:**
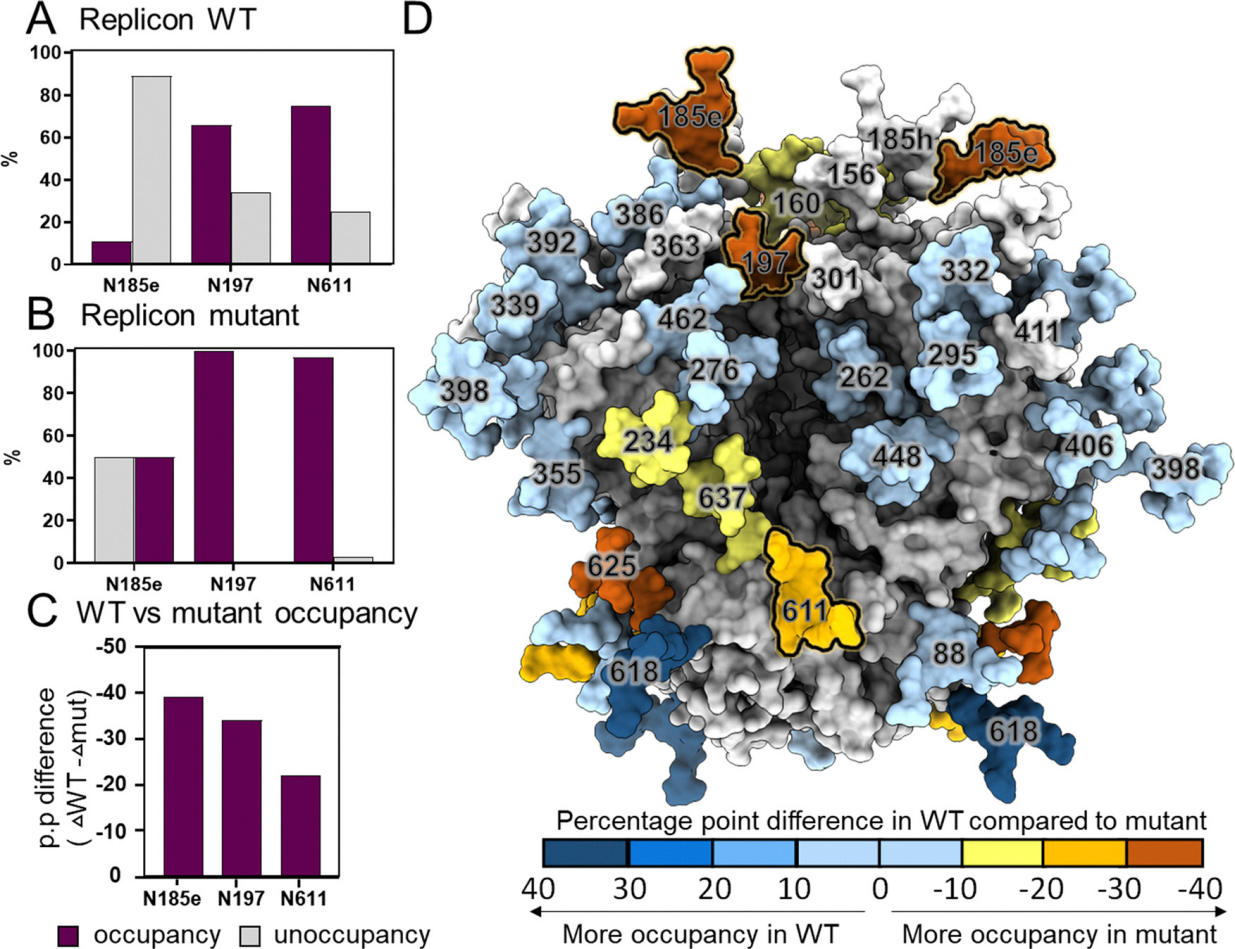
Changes in glycan occupancy by nucleotide editing. (A) Bar graph representing glycan occupancy on wildtype Env, (B) mutant Env with NXS to NXT mutation and (C) wildtype *versus* mutant Env. The three sites, N185e, N197, N611 which were NXS on wildtype have been edited to NXT. The percentage glycan occupancy is shown in violet and unoccupancy at glycan sites in gray. (D) A model of HIV-1 Env based upon PDB ID 5C7K, mapped with the percentage point differences in glycan occupancy in WT Env compared to mutant Env. Not determined glycan sites are shown in white.

We sequentially mutated each site and incorporated all three site-specific mutations in a single plasmid for transcription of RNA expressing altered Env, which we are referring as the ‘mutant’. The RNA construct with the original, unaltered gene sequence for the BG505 NFL.664 Env is designated here as the 'wild-type' (WT). Firstly, we performed NS-EM of the replicon expressed mutant Env to determine the trimeric assembly of the Env. The NS-EM data of the mutant Env revealed the presence of well-formed native like trimers, however the proportion of misfolded material was higher compared to the WT NFL Env (Fig. S6). Furthermore, we performed site-specific analysis on the mutated Env protein and compared it with the WT Env to determine any improvement in glycan occupancy. The N197 and N611 sites were less occupied on WT Env but when altered, are almost fully occupied (Fig. 5A and B). Similarly, the N185e site contains mostly unoccupied glycans (∼90%) on WT Env. However, when serine adjacent to the *N*-glycan site was substituted with threonine, the glycan occupancy was restored to 50% (Fig. 5A and B).

We then compared the percentage point change in glycan composition of WT with mutant and observed a significant elevation in glycan occupancy at all three glycan sites compared to WT Env (Fig. 5C and D). Furthermore, to explore the effect of these mutations on all the PNGS including these edited sites, we mapped the percentage point changes in glycan occupancy observed on WT Env compared to mutant Env (Fig. 5D). We observed that N625, N637 and N234 which are near the altered site N611, are more occupied on mutated Env protein compared to WT (Fig. 5D). However, the N618, which is adjacent to the N625 site, decreased in occupancy on mutated Env are more occupied compared to WT Env that display low glycan occupancy.

Furthermore, to determine the binding of the WT and mutant Env, we performed ELISAs with selected bnAbs and non-nAbs. Overall, the binding was comparable between WT and mutant Env with the bnAbs, VRC01, PGT121 and 2G12 (Fig. S7A–C). Both WT and mutant Env exhibited negligible binding with non-nAb F240 (Fig. S7D). The non-nAb RM20E1, which binds to the Env lacking glycan at N611, showed binding with the unmutated Env with underoccupied N611 sites whereas not with the mutant Env displaying full occupancy at N611. This further confirms the restoration of glycan occupancy at N611 site of mutant Env. The targeted repair of Env can be used to improve the glycan occupancy of the RNA-based immunogens, however other factors need to considered such as assembly of the Env and off-target antibody responses.

The data presented here corresponds to soluble HIV-1 Env which presents near native-like complex-type and oligomannose-type glycan sites but with a few *N*-linked glycosylation sites showing low occupancy. In contrast, full-length membrane-bound HIV-1 Env, currently favored for nucleotide-based vaccines, is less likely to exhibit glycan holes.^52,64^ Similarly, the cell lines utilized in this study do not fully recapitulate the complex cellular and tissue landscape at the site of injection in humans. We expect that replicon-derived Env would exhibit a range of different glycoforms representing the individual cell types transfected in a immunization setting. Overall, this study emphasizes the significance of considering glycan site occupancy and glycan heterogeneity when developing and assessing vaccine candidates, particularly based on the HIV-1 Env glycoprotein.

## Materials and methods

### Replicon production and purification

RNA replicon was derived from alphavirus VEE's genome as described previously.^90^ The VEEV genome is modified, and the structural genes are replaced with Env gene, BG505 NFL.664 (PDB: 6P62). Briefly, plasmid DNA encoding for replicon was grown in *Escherichia coli* and purified using the Plasmid QIAprep spin maxi kit (QIAGEN). The plasmid backbone sequence contains the Ampicillin marker, pBR322 ori, nsp1-4 sequons of VEE's genome. The replicon sequence is flanked by the T7 promoter on the 5′ end and MluI restriction sites at 5′ end of the T7 promoter and 3′ end encoded poly(A) sequence. To generate the template for *in vitro* transcription (IVT) reaction, first the plasmid DNA was linearized *via* the digestion of these plasmids using MluI (Thermo Fisher Scientific) and cleaned using the DNA purification kit (QIAGEN). Linearized DNAs were then transcribed using mMessage IVT kit and reaction was set up according to manufacturer's protocol. The RNA was then cleaned using megaclear kit (Thermo Fisher Scientific) to remove any buffers or impurities from the IVT reaction. Before transfection, RNA was run on a gel using RNA gel electrophoresis to check the purity of RNA. The RNA concentration and purity was verified by spectrophotometry using NanoDrop One and was then stored at −80 °C until further use.

### Culturing of mammalian cell lines

HEK293F cells were cultured in freestyle media and are maintained at a density of 0.2 × 10^6^ cells per mL at 37 °C, 8% CO_2_, 125 rpm. The cells were transfected at a density of 1 × 10^6^ cells per mL.

The mouse muscle cell line (C2C12) was cultured in Dulbecco's modified Eagles medium supplemented with 10% fetal bovine serum (FBS) as recommended in manufacturer's protocol (American type culture collection, Catalogue no. CRL-1772). The cells were maintained in T75 adherent flasks at 2 × 10^6^ cells per mL at 37 °C, 5% CO_2_.

The dendritic cell lines (DC2.4) was maintained in RPMI-1640 media supplemented with 10% FBS, l-glutamine, non-essential amino acids, HEPES buffer, and β-mercaptoethanol according to manufacturer's protocol (Sigma-Aldrich, Catalogue no. SCC142). The cells were maintained in T75 adherent flasks at 2 × 10^6^ cells per mL at 37 °C, 5% CO_2_.

### *In vitro* transfection of RNA replicon

To assess the assembly and glycosylation of Env protein HEK cells were transfected with RNA. Further to investigate the cell derived changes in replicon expression of Env, C2C12 and DC2.4 were transfected with RNA. The RNA replicon transfection was carried out using two methods, neon transfection system (Thermo Fisher Scientific) and TransIT-mRNA kit (Mirus Bio). Using the neon transfection system, cells were electroporated with RNA using 100 µL neon tip, according to manufacturer's protocol. Briefly, the required number of the cells were pelleted and washed with PBS, then cells were prepared for electroporation, by mixing 1 µg RNA per 1 × 10^6^ cells in serum free media, OptiMem. Followed by electroporation at 1200 V, 30 ms and then the cells were ejected in 6 well plates containing 2 mL media for culturing of cells. Each mL contains electroporated solution of 1 × 10^6^ cells mixed with 1 µg RNA. The supernatant was harvested after 48 h and supplemented with new media and harvested again after 24 h to attain high yield of protein expression.

Using the TransIT-mRNA kit, transfection was carried out as directed in manufacturer's protocol. Briefly, suspension HEK 293F cells were grown to 1–1.5 × 10^6^ cells per mL in 25 mL Freestyle media. The adherent cells were seeded at 2 × 10^6^ cells in 12 mL of media in T75 flask and were transfected at 80% confluency. The transfection solution was composed of 100 µL of Reduced sera free media, OptiMem, 1 µg of RNA replicon, 2 µL of the mRNA boost reagent and 2 µL of the Trans-IT mRNA reagent for 1 mL of seeded cells. The transfection mixture was then incubated at room temperature for 2–5 min. Followed by addition of transfection complexes dropwise to the cells. The cells were incubated at 37 °C for 24 h for suspension cells and 48 h for adherent cells. The supernatant was harvested after incubation and the cells were pelleted down, followed by filtering of the supernatant *via* 0.22 µm pore size sterile filter.

### Transient transfection of plasmid DNA

Plasmid encoding BG505 NFL.664 Env protein was used to transiently transfect HEK 293F cells using polyethyleneimine (PEI). The plasmid DNA contains the backbone of pcDNA 3.1 (-) and the BG505 NFL.664 gene was inserted between XbaI and HindIII restriction sites as described previously.^39^ Briefly, the cells were maintained at a density maintained at a density of 0.2–3.0 × 10^6^ cells per mL at 37 °C, 8% CO_2_ and 125 rpm. Transfection mix was prepared in Opti-MEM using two solutions, DNA (310 µg L^−1^) and PEI max reagent (1 mg mL^−1^, pH 7) in a ratio of 1 : 3 in 25 mL of Opti-MEM respectively, followed by addition of DNA solution to the PEI mix and incubated for 20 min at room temperature. Cells were transfected at a density of 1 × 10^6^ cells per mL and incubated at 37 °C, 8% CO_2_ and 125 rpm. Culture was harvested after 7 days post transfection and the media was separated from the cells by centrifugation at 4000 rpm for 30 min. The supernatant was filtered using 0.22 µm pore size sterile filter.

### Antibody expression

Plasmid encoding heavy and light chains of PGT145 antibody were transfected in HEK 293F cells using PEI. Transfection mix was prepared in OptiMeM using three solution, heavy chain DNA (155 µg L^−1^), light chain (155 µg L^−1^), and DNA PEI max reagent (1 mg mL^−1^, pH 7) in a ratio of 1 : 3 in 25 mL of Opti-MEM respectively, followed by addition of DNA solution to the PEI mix and incubated for 20 min at room temperature. The cells were cultured and harvested in same way as described above. The supernatant was then purified using protein A affinity chromatography.

### Env purification

The supernatant containing soluble Env protein was collected and purified by affinity chromatography *Galanthus nivalis* lectin (GNL) beads (Vector Labs) according to manufacturer's protocol. Briefly, 10 column volumes of wash buffer, 1 × PBS was used to wash the beads and then supernatant containing Env was applied, and the solution was allowed to drain through gravity. After application of sample, the beads were washed again with 3 columns of wash buffer to remove unbound materials. Lastly, the protein was eluted using 3–5 columns of elution buffer, 0.5 M α-methylmannoside, pH 4. The eluted protein was then concentrated using 100 kDa Amicon filter.

For purification of trimer specific Env protein, the supernatant containing Env was purified by PGT145 antibody.^91^ The antibody was added at the concentration of 10 µg mL^−1^ to the supernatant, the mixture was incubated overnight at 4 °C. Followed by purification of PGT145 bound trimer specific Env using protein A affinity chromatography.

### Negative-stain electron microscopy and 2D class averaging

3 µL of protein at 0.02 mg mL^−1^ was applied to a 400 mesh Cu grid, blotted with filter paper, and stained with 2% uranyl formate. Micrographs were collected on a Thermo Fisher Tecnai Spirit microscope operating at 120 kV with a FEI Eagle CCD (4k) camera (2.06 Å per pixel; 52 000 × magnification) using Leginon automated image collection software.^92^ Particles were picked using DogPicker^93^ and 2D classification was processed with iterative multivariate statistical analysis (MSA)/multireference alignment (MRA).^94^

### Glycopeptide analysis by mass spectrometry

Env proteins were denatured for 1 h in 50 mM Tris/HCl, pH 8.0 containing 6 M of urea. Next, the sample was reduced and alkylated by adding 5 mM dithiothreitol (DTT) and 20 mM iodoacetamide (IAA) and incubated for 1 h in the dark, followed by a 1 h incubation with 20 mM DTT to eliminate residual IAA. The alkylated Env glycoproteins were buffer exchanged into 50 mM Tris/HCl, pH 8.0 using Vivaspin columns (3 kDa) and three aliquots were digested separately overnight using trypsin (Mass Spectrometry grade, Promega), chymotrypsin (Mass Spectrometry Grade, Promega) or alpha lytic protease (Sigma Aldrich) at a ratio of 1 : 30 (w/w) at 37 °C. The next day, the peptides were dried and extracted using C18 Zip-tip (MerckMilipore) or Oasis HLB cartridges (Waters) according to manufacturer's protocol. Following the extraction, the peptides were dried again, re-suspended in 0.1% formic acid, and analyzed by nanoLC-ESI MS with an Ultimate 3000 high-performance liquid chromatography (HPLC) (Thermo Fisher Scientific) system coupled to an Orbitrap Eclipse mass spectrometer (Thermo Fisher Scientific) using stepped higher energy collision-induced dissociation (HCD) fragmentation. Peptides were separated using an EasySpray PepMap RSLC C18 column (75 µm × 75 cm). A trapping column (PepMap 100 C18 3 µm particle size, 75 µm × 2 cm) was used in line with the LC prior to separation with the analytical column. The LC conditions were as follows: 280-minute linear gradient consisting of 4–32% acetonitrile in 0.1% formic acid over 260 min followed by 20 min of alternating 76% acetonitrile in 0.1% formic acid and 4% ACN in 0.1% formic acid, used to ensure all the sample had eluted from the column. The flow rate was set to 300 nL min^−1^. The spray voltage was set to 2.5 kV and the temperature of the heated capillary was set to 40 °C. The ion transfer tube temperature was set to 275 °C. The scan range was 375–1500 *m*/*z*. Stepped HCD collision energy was set to 15, 25 and 45% and the MS2 for each energy was combined. Precursor and fragment detection were performed using an Orbitrap at a resolution MS1 = 120 000. MS2 = 30 000. The automatic gain control (AGC) target for MS1 was set to standard and injection time set to auto which involves the system setting the two parameters to maximize sensitivity while maintaining cycle time. Full LC and MS methodology can be extracted from the appropriate Raw file using XCalibur FreeStyle software.

Glycopeptide fragmentation data were extracted from the raw file using Byos (Version 4.0; Protein Metrics Inc.). The glycopeptide fragmentation data were evaluated manually for each glycopeptide; the peptide was scored as true-positive when the correct b and y fragment ions were observed along with oxonium ions corresponding to the glycan identified. The MS data was searched using the Protein Metrics 305 *N*-glycan library with sulfated glycans added manually. The relative amounts of each glycan at each site as well as the unoccupied proportion were determined by comparing the extracted chromatographic areas for different glycotypes with an identical peptide sequence. All charge states for a single glycopeptide were summed. The precursor mass tolerance was set at 4 ppm and 10 ppm for fragments. A 1% false discovery rate (FDR) was applied. The relative amounts of each glycan at each site as well as the unoccupied proportion were determined by comparing the extracted ion chromatographic areas for different glycopeptides with an identical peptide sequence. Glycans were categorized according to the composition detected as listed in Fig. 4.

### Site-directed mutagenesis of BG505 NFL.664 Env

To improve the occupancy, threonine substitutions were introduced into the env gene leading to NXS to NXT transition at *N*-linked glycan sites, N185e, N197, and N611. We sequentially mutated these sites by three separate polymerase chain reactions (PCRs) using three sets of forward and reverse primers carrying mutations for different sites. We designed the primers using Agilent quick change primer design manufacturer's PCR conditions (Thermo Fisher Phusion polymerase) with 10 ng µL^−1^ env gene in a final volume of 20 µL. The reaction was then set on thermal cycler at 98 °C initial denaturation and then 18 cycles with set conditions as 98 °C for 20 seconds, 58–68 °C for 30 seconds, 72 °C for 6 min, followed by final extension at 72 °C for 5 min. The PCR product was digested with dpnI, followed by transformation with XL-10 gold cells. The plasmid DNA was sequenced and subsequently used in the PCR reactions for substitution of all three sites.

### Model construction

The structural models used for presentation of *N*-linked glycan of BG505 NFL.664 is modelled on BG505 SOSIP.664 Env structure (PDB ID: 5C7K) as both of the structures are highly similar and superimpose on each other.^40,68^

## Author contributions

H. C., R. J. S., A. B. W., D. J. I. and M. C. conceived the research and designed the experiments. R. J. S., A. B. W., D. J. I. and M. C. supervised the research experiments. H. C., J. B., J. T. W. and G. M. H. performed the RNA preparation and protein expression. H. C. performed mass spectrometry experiments and analyzed data. M. S. and D. J. I. provided the replicon constructs. W. L., G. O. and A. B. W. performed and analyzed the NS-EM data. P. F. M. and R. J. S. helped with initial set up of RNA preparations. H. C. and M. C. wrote the original draft of the manuscript. All authors commented upon and approved the final manuscript.

## Conflicts of interest

The authors declare that there are no conflicts of interest.

## Supplementary Material

CB-OLF-D5CB00165J-s001

## Data Availability

Mass spectrometry raw files have been deposited in the MassIVE proteomics database (MSV000091334). Supplementary information (SI) is available. See DOI: https://doi.org/10.1039/D5CB00165J.
