# Supplementary material for: Signatures of native-like glycosylation in RNA replicon-derived HIV-1 immunogens
Source: RSC Chem Biol. 2026 Jan 12;7(3):400–13. doi: 10.1039/d5cb00165j (PMC12853648; doi:10.1039/d5cb00165j)
Supplement: CB-007-D5CB00165J-s001 [file CB-007-D5CB00165J-s001.pdf]

Supplementary Information (SI) for RSC Chemical Biology.
This journal is © The Royal Society of Chemistry 2026

Supplementary Information for:

Signatures of native-like glycosylation in RNA replicon-derived HIV-1 immunogens

Himanshi Chawla[1], Jacob T. Willcox[1], Grace M. Hayes[1], Murillo Silva[2], Wen-Hsin Lee[3,4], Gabriel Ozorowski[3,4], John Butler[1], Paul F. Mckay[5], Robin J. Shattock[5], Andrew B. Ward[3,4], Darrell J. Irvine[2,4], Max Crispin[1*]

[1]School of Biological Sciences, University of Southampton, Southampton SO17 1BJ, UK

[2]Koch Institute for Integrative Cancer Research, Massachusetts Institute of Technology, Cambridge, MA, USA

[3]Department of Integrative Structural and Computational Biology, The Scripps Research Institute, La Jolla, CA 92037, USA

[4]Center for HIV/AIDS Vaccine Development, IAVI Neutralizing Antibody Center and the Collaboration for AIDS Vaccine Discovery (CAVD), The Scripps Research Institute, La Jolla, CA 92037, USA

[5]Department of Infectious Diseases, Imperial College London, Norfolk Place, London, W2 1PG, UK

*Corresponding author. Email: max.crispin@soton.ac.uk

This document includes

Supplementary Tables 1 to 4

Supplementary Figure 1 to 7

Supplementary Table 1: Glycan composition analysis of replicon expressed env in HEK 293F cells. The upper table shows the categorized glycan compositions at each N-linked glycan site with the reported value the mean of three or more biological replicates. The global averages are shown in the right-hand table. The lower table further categorizes the glycan compositions into oligomannose-, hybrid-, and complex-type as well as the percentage of glycan compositions containing at least one fucose or one sialic acid residue and core-type glycan structures.

	N88	N133	N142	N156	N160	N185e	N185h	N197	N234	N262	N276	N295	N301	N332	N339	N355	N363	N386	N392	N398	N406	N411	N448	N462	N611	N618	N625	N637
M9Glc	0	0	0	0	0	0	0	0	0	0	0	0	0	0	0	0	0	0	0	0	0	0	0	0	0	0	0	0
M9	0	0	0	78	24	0	0	13	26	39	0	86	0	72	14	0	38	42	28	0	1	1	30	0	0	0	0	1
M8	0	7	0	0	24	0	0	10	25	22	14	8	0	22	31	0	18	22	34	0	5	7	20	2	1	0	0	3
M7	2	0	0	22	10	0	0	3	11	9	15	0	0	3	12	1	7	12	10	0	5	19	8	1	2	0	0	21
M6	2	6	0	0	9	0	0	4	5	6	7	0	0	2	6	1	3	9	7	0	6	13	5	1	1	0	0	11
M5	11	21	0	0	9	2	1	10	9	15	29	6	0	1	6	42	7	10	11	3	34	23	9	6	14	10	0	17
M4	0	14	0	0	1	0	0	1	0	2	3	0	0	0	2	2	1	2	2	1	10	4	3	2	1	0	0	2
M3	0	0	0	0	1	0	0	0	0	0	1	0	0	1	0	1	0	1	1	0	0	2	3	0	0	0	0	3
FM	0	0	0	0	0	0	4	0	0	0	0	0	0	0	0	0	0	0	0	0	0	0	0	0	0	2	9	0
Hybrid	1	0	66	0	1	0	0	1	0	0	5	0	0	0	1	10	1	1	0	0	7	10	3	0	1	0	1	6
Fhybrid	0	0	0	0	0	0	0	2	0	0	0	0	0	0	0	3	1	0	0	1	4	0	0	0	1	0	0	3
HexNAc(3)(x)	3	0	6	0	0	0	0	0	0	0	5	0	0	0	0	0	0	0	0	0	4	0	1	1	3	0	1	0
HexNAc(3)(F)(x)	1	27	0	0	2	1	0	2	0	0	2	0	0	0	1	4	1	0	0	11	6	2	1	5	2	0	0	2
HexNAc(4)(x)	18	0	23	0	0	0	3	0	0	0	1	0	0	0	0	0	0	0	0	12	2	0	1	0	0	0	0	0
HexNAc(4)(F)(x)	27	0	4	0	2	3	0	12	7	5	7	0	0	1	2	15	5	0	2	52	14	0	7	46	10	25	1	12
HexNAc(5)(x)	8	0	0	0	0	0	82	0	0	0	0	0	0	0	0	0	0	0	0	0	0	0	0	0	0	0	0	0
HexNAc(5)(F)(x)	9	7	1	0	2	2	1	5	1	1	6	0	0	0	1	14	2	0	1	17	2	0	3	26	24	9	0	5
HexNAc(6+)(x)	0	0	0	0	0	0	0	0	0	0	0	0	0	0	0	0	0	0	0	0	0	0	0	0	0	0	0	0
HexNAc(6+)(F)(x)	7	18	0	0	0	0	7	1	0	0	3	0	0	0	0	2	0	0	0	1	0	0	0	5	13	5	0	1
UNOCC	9	0	0	0	15	89	0	34	12	0	0	0	0	0	21	4	14	0	0	0	0	17	1	0	25	50	87	14
core	1	0	0	0	2	1	3	1	3	2	1	0	0	0	2	0	1	2	4	0	0	1	7	4	3	0	0	1

	Total
M9Glc	0
M9	18
M8	10
M7	6
M6	4
M5	11
M4	2
M3	1
FM	1
Hybrid	4
Fhybrid	1
HexNAc(3)(x)	1
HexNAc(3)(F)(x)	3
HexNAc(4)(x)	2
HexNAc(4)(F)(x)	9
HexNAc(5)(x)	3
HexNAc(5)(F)(x)	5
HexNAc(6+)(x)	0
HexNAc(6+)(F)(x	2
Unoccupied	14
core	1

%	N88	N133	N142	N156	N160	N185e	N185h	N197	N234	N262	N276	N295	N301	N332	N339	N355	N363	N386	N392	N398	N406	N411	N448	N462	N611	N618	N625	N637
Mannose	15	47	0	100	77	3	5	41	77	92	69	100	0	99	72	48	75	97	93	5	61	70	77	11	19	12	10	58
Hybrid	1	0	66	0	1	0	0	3	0	0	5	0	0	0	1	13	2	1	0	1	10	10	3	1	1	0	1	8
Complex	73	53	34	0	5	6	93	20	8	6	25	0	0	1	3	35	8	1	3	94	29	3	12	84	52	39	2	19
Unoccupied	9	0	0	0	15	89	0	34	12	0	0	0	0	0	21	4	14	0	0	0	0	17	1	0	25	50	87	14
Fucosylation	44	53	5	0	5	6	12	21	9	6	19	0	0	1	4	38	9	1	3	83	26	2	11	83	50	40	10	22
Sialylation	25	8	93	0	0	1	10	6	4	3	10	0	0	0	0	13	0	0	0	57	12	1	2	33	9	9	0	4
core	1	0	0	0	2	1	3	1	3	3	0	0	0	0	2	0	1	2	4	0	0	1	6	4	3	0	0	1

	Total
Mannose	51
Hybrid	5
Complex	29
Unoccupied	14
Fucosylation	21
Sialylation	11
core	1

Supplementary Table 2: Glycan composition analysis of replicon expressed Env in C2C12 cells.

	N88	N133	N142	N156	N160	N185e	N185h	N197	N234	N262	N276	N295	N301	N332	N339	N355	N363	N386	N392	N398	N406	N411	N448	N462	N611	N618	N625	N637
M9Glc	0	0	0	0	0	0	0	0	0	4	0	0	0	0	0	0	0	0	0	0		0	0	0	0	0	0	0
M9	0	16	0	66	14	0	0	15	66	48	2	81	0	90	54	0	54	81	46	0		0	42	0	0	0	0	3
M8	0	22	0	34	38	0	0	17	29	25	22	13	0	10	17	10	8	9	44	0		42	30	0	0	0	19	23
M7	0	5	0	0	45	1	0	19	2	3	34	1	0	0	3	28	3	2	3	0		11	11	1	1	0	17	31
M6	0	6	0	0	3	2	0	13	0	5	8	0	0	0	4	34	1	1	2	0		45	9	13	27	0	63	30
M5	12	5	0	0	0	0	0	6	1	7	15	0	3	0	1	14	1	2	3	0		1	6	9	7	0	0	2
M4	0	0	0	0	0	0	0	0	0	1	4	0	0	0	1	1	0	0	2	0		0	0	0	0	0	0	0
M3	0	0	0	0	0	0	0	0	0	0	1	0	0	0	0	0	0	0	0	0		0	1	0	0	0	0	0
FM	0	0	0	0	0	0	0	0	0	0	0	0	0	0	0	0	0	0	0	0		0	0	0	0	0	0	0
Hybrid	48	25	0	0	0	0	0	1	0	0	0	0	0	0	0	1	0	0	0	0		0	0	0	0	0	0	3
Fhybrid	0	0	0	0	0	0	0	0	0	0	0	0	0	0	0	0	0	0	0	0		0	0	0	0	0	0	0
HexNAc(3)(x)	1	0	0	0	0	0	0	0	0	0	0	0	0	0	0	0	0	0	0	0		0	0	0	0	0	0	0
HexNAc(3)(F)(x)	0	0	0	0	0	0	0	0	0	0	0	0	0	0	0	0	0	0	0	0		0	0	0	0	0	0	0
HexNAc(4)(x)	36	0	77	0	0	0	1	0	0	0	0	0	97	0	0	0	0	0	0	0		0	0	0	1	1	0	0
HexNAc(4)(F)(x)	3	0	0	0	0	1	0	6	2	3	3	0	0	0	0	9	2	0	0	99		0	1	37	59	99	0	0
HexNAc(5)(x)	0	0	0	0	0	0	0	0	0	0	0	0	0	0	0	0	0	0	0	0		0	0	0	0	0	0	0
HexNAc(5)(F)(x)	0	20	0	0	0	0	0	0	0	0	0	6	0	0	0	1	0	0	0	1		0	0	31	6	0	0	0
HexNAc(6+)(x)	0	0	0	0	0	0	0	0	0	0	0	0	0	0	0	0	0	0	0	0		0	0	0	0	0	0	0
HexNAc(6+)(F)(x)	0	0	23	0	0	0	99	0	0	0	0	0	0	0	0	0	0	0	0	0		0	0	0	0	0	0	0
UNOCC	0	0	0	0	0	96	0	22	0	3	8	0	0	0	19	0	30	5	0	0		0	0	0	0	0	0	7
core	0	0	0	0	0	0	0	0	0	0	4	0	0	0	1	0	0	0	0	0		0	0	9	0	0	0	0

Total	
M9Glc	0
M9	25
M8	15
M7	8
M6	10
M5	4
M4	0
M3	0
FM	0
Hybrid	3
Fhybrid	0
HexNAc(3)(x)	0
HexNAc(3)(F)(x)	0
HexNAc(4)(x)	8
HexNAc(4)(F)(x)	12
HexNAc(5)(x)	0
HexNAc(5)(F)(x)	2
HexNAc(6+)(x)	0
HexNAc(6+)(F)(x)	5
Unoccupied	7
core	1

%	N88	N133	N142	N156	N160	N185e	N185h	N197	N234	N262	N276	N295	N301	N332	N339	N355	N363	N386	N392	N398	N406	N411	N448	N462	N611	N618	N625	N637
Mannose	12	55	0	100	100	3	0	69	98	94	85	94	3	100	80	87	67	95	100	0	0	99	98	23	34	0	100	90
Hybrid	48	25	0	0	0	0	0	2	0	0	0	0	0	0	0	1	1	0	0	0	0	0	0	0	0	0	0	3
Complex	40	20	100	0	0	1	100	7	2	4	3	6	97	0	0	11	2	0	0	100	0	0	1	68	66	100	0	0
Unoccupied	0	0	0	0	0	96	0	22	0	3	8	0	0	0	19	0	30	5	0	0	0	1	1	0	0	0	0	7
Fucosylation	3	20	23	0	0	1	99	7	2	4	3	6	0	0	0	11	2	0	0	100	0	0	2	68	65	99	0	0
Sialylation	73	45	23	0	0	1	0	2	0	4	3	0	97	0	0	10	1	0	0	100	0	0	0	8	62	99	0	0
core	0	0	0	0	0	0	0	0	0	0	4	0	0	0	1	0	0	0	0	0	0	0	0	9	0	0	0	0

Total	
Mannose	60
Hybrid	3
Complex	26
Unoccupied	7
Fucosylation	18
Sialylation	19
core	0

Supplementary Table 3: Glycan composition analysis of replicon expressed Env in DC2.4 cells

	N88	N133	N142	N156	N160	N185e	N185h	N197	N234	N262	N276	N295	N301	N332	N339	N355	N363	N386	N392	N398	N406	N411	N448	N462	N611	N618	N625	N637
M9Glc	0		0	0	0	0	0	0	0	0	0	0	0	0	0	0	0	0	0	0	0	0	0	0	0	0	0	0
M9	0		0	0	61	0	0	2	47	15	0	26	0	69	41	0	64	92	51	0	0	3	38	2	0	0	0	0
M8	0		0	0	0	0	0	4	20	57	59	7	0	10	18	3	16	5	44	0	0	7	26	0	0	0	0	2
M7	1		0	0	0	1	0	4	2	1	9	0	0	2	3	4	2	1	0	0	0	10	3	5	0	0	0	6
M6	4		13	13	39	0	0	2	1	0	11	22	0	0	1	34	0	1	0	0	0	11	1	0	0	0	0	5
M5	15		0	87	0	1	0	4	1	20	0	35	0	16	1	14	2	1	1	0	100	53	10	14	2	0	0	4
M4	0		0	0	0	0	0	0	0	0	0	0	0	4	0	1	0	0	0	0	0	0	0	0	0	0	0	0
M3	7		0	0	0	0	0	0	0	0	0	0	0	0	0	0	0	0	0	0	0	0	0	0	0	0	0	0
FM	0		0	0	0	0	0	0	0	0	0	0	0	0	0	0	0	0	0	0	0	0	0	0	0	0	0	0
Hybrid	32		0	0	0	0	0	1	0	0	1	0	0	0	0	8	0	0	4	0	0	2	0	0	0	0	0	16
Fhybrid	0	N.D.	0	0	0	0	0	0	0	0	0	0	0	0	0	1	0	0	0	0	0	0	0	0	0	0	0	0
HexNAc(3)(x)	0		0	0	0	0	0	0	0	0	0	10	0	0	0	0	0	0	0	0	0	0	0	0	0	0	0	0
HexNAc(3)(F)(x)	0		0	0	0	0	0	0	1	0	0	0	0	0	0	2	0	0	0	1	0	0	0	0	0	0	0	12
HexNAc(4)(x)	23		0	0	0	0	0	0	2	0	0	0	67	0	0	7	1	0	0	0	0	0	0	0	0	4	0	0
HexNAc(4)(F)(x)	5		0	0	0	0	0	2	26	0	20	0	0	0	0	15	0	0	0	99	0	0	0	62	0	21	0	1
HexNAc(5)(x)	0		50	0	0	0	0	0	1	0	0	0	33	0	0	0	0	0	0	0	0	0	0	0	0	0	0	0
HexNAc(5)(F)(x)	0		0	0	0	0	0	0	0	0	0	0	0	0	0	7	0	0	0	0	0	0	14	0	25	0	0	18
HexNAc(6+)(x)	0		3	0	0	0	0	0	0	0	0	0	0	0	0	0	0	0	0	0	0	0	0	0	0	0	0	0
HexNAc(6+)(F)(x)	0		34	0	0	0	100	0	0	0	0	0	0	0	0	0	0	0	0	0	0	0	0	0	0	75	0	4
UNOCC	11		0	0	0	94	0	80	0	7	0	0	0	0	34	5	15	0	0	0	0	14	8	0	72	0	100	33
core	0		0	0	0	3	0	0	0	0	0	0	0	0	0	0	0	0	0	0	0	0	0	17	0	0	0	0

Total	
M9Glc	0
M9	19
M8	10
M7	2
M6	6
M5	14
M4	0
M3	0
FM	0
Hybrid	2
Fhybrid	0
HexNAc(3)(x)	0
HexNAc(3)(F)(x)	1
HexNAc(4)(x)	4
HexNAc(4)(F)(x)	9
HexNAc(5)(x)	3
HexNAc(5)(F)(x)	2
HexNAc(6+)(x)	0
HexNAc(6+)(F)(x)	8
Unoccupied	18
core	1

%	N88	N133	N142	N156	N160	N185e	N185h	N197	N234	N262	N276	N295	N301	N332	N339	N355	N363	N386	N392	N398	N406	N411	N448	N462	N611	N618	N625	N637
Mannose	28		13	100	100	2	0	17	71	93	79	90	0	100	65	56	85	100	96	0	100	84	77	21	3	0	0	16
Hybrid	32		0	0	0	0	0	1	0	0	1	0	0	0	0	8	0	0	4	0	0	2	0	0	0	0	0	16
Complex	29		87	0	0	0	100	2	29	0	20	10	100	0	0	31	1	0	0	100	0	0	14	62	25	100	0	35
Unoccupied	11	N.D.	0	0	0	93	0	80	0	7	0	0	0	0	34	5	15	0	0	0	0	14	8	0	72	0	100	33
Fucosylation	5		34	0	0	0	100	2	26	0	20	0	0	0	0	25	0	0	0	100	0	0	14	62	25	96	0	35
Sialylation	60		37	0	0	0	0	0	15	0	11	0	66	0	0	22	1	0	4	50	0	2	0	19	25	0	0	34
core	0		0	0	0	0	0	0	0	0	0	0	0	0	0	0	0	0	0	0	0	0	0	7	0	0	0	0

Total	
Mannose	52
Hybrid	2
Complex	28
Unoccupied	17
Fucosylation	20
Sialylation	13
core	0

Supplementary Table 4: Glycan composition analysis of transiently expressed Env via plasmid DNA in HEK 293F cells

	N88	N133	N142	N156	N160	N185e	N185h	N197	N234	N262	N276	N295	N301	N332	N339	N355	N363	N386	N392	N398	N406	N411	N448	N462	N611	N618	N625	N637
M9Glc	0	0			0	0	0	0	0	0	0	0		0	0	0	0	0	0	0	0	0	0	0	0	0	0	0
M9	0	0			30	0	0	16	45	36	0	41		71	42	0	54	47	25	0	0	0	27	0	0	0	0	0
M8	0	0			17	0	0	11	25	32	16	17		19	27	0	11	19	43	0	0	4	25	0	0	0	0	6
M7	0	0			7	0	0	5	13	7	15	10		5	10	1	9	9	9	0	0	10	10	0	0	0	0	19
M6	1	0			3	0	0	5	5	9	8	23		2	5	1	6	8	8	0	0	13	5	0	0	0	0	18
M5	17	0			30	0	0	16	5	5	25	9		2	6	24	12	13	12	0	100	57	16	1	6	0	0	25
M4	0	0			2	0	0	0	0	1	9	0		0	2	1	1	1	1	0	0	1	3	0	0	0	0	2
M3	0	0			0	0	0	0	0	0	1	0		0	0	0	0	0	0	0	0	0	1	0	0	0	0	0
FM	0	0			0	0	0	0	0	0	0	0		0	0	1	0	0	0	0	0	0	0	0	0	0	0	0
Hybrid	1	0			1	0	0	0	0	0	14	0		0	0	7	2	1	0	0	0	8	3	0	0	0	5	11
Fhybrid	0	0			0	0	0	0	0	0	0	0		0	0	10	1	0	0	1	0	1	1	0	0	0	0	2
HexNAc(3)(x)	1	0			0	0	0	0	0	0	1	0		0	0	1	0	0	0	0	0	2	0	0	0	0	1	0
HexNAc(3)(F)(x)	1	37			0	1	0	3	0	0	0	0		0	0	14	2	0	0	6	0	1	1	1	0	0	0	1
HexNAc(4)(x)	11	0			0	0	100	0	0	0	2	0		0	0	0	0	0	0	0	0	0	0	0	0	0	0	0
HexNAc(4)(F)(x)	15	63			3	3	0	10	2	6	5	0		0	1	20	1	0	0	28	0	1	6	45	4	6	0	7
HexNAc(5)(x)	24	0			0	0	0	0	0	0	1	0		0	0	0	0	0	0	0	0	0	0	0	0	0	1	0
HexNAc(5)(F)(x)	20	0			0	5	0	3	0	3	1	0		0	0	16	1	0	0	37	0	0	2	42	33	2	0	2
HexNAc(6+)(x)	1	0			0	0	0	0	0	0	0	0		0	0	0	0	0	0	29	0	0	0	0	0	0	0	0
HexNAc(6+)(F)(x)	1	0			0	1	0	0	0	0	2	0		0	0	2	0	0	0	0	0	0	0	9	31	0	0	2
UNOCC	3	0			5	88	0	30	2	0	0	0		0	5	0	0	0	0	0	0	0	0	0	24	92	92	4
core	6	0			1	0	0	1	1	1	1	0		0	0	2	0	0	0	0	0	1	0	2	1	0	0	1

Total	
M9Glc	0
M9	17
M8	11
M7	6
M6	5
M5	15
M4	1
M3	0
FM	0
Hybrid	2
Fhybrid	1
HexNAc(3)(x)	0
HexNAc(3)(F)(x)	3
HexNAc(4)(x)	5
HexNAc(4)(F)(x)	9
HexNAc(5)(x)	1
HexNAc(5)(F)(x)	7
HexNAc(6+)(x)	1
HexNAc(6+)(F)(x)	2
UNOCC	14
core	1

%	N88	N133	N142	N156	N160	N185e	N185h	N197	N234	N262	N276	N295	N301	N332	N339	N355	N363	N386	N392	N398	N406	N411	N448	N462	N611	N618	N625	N637
Mannose	18	0			90	1	0	53	94	89	74	100		99	93	27	93	98	99	0	100	86	87	1	6	0	0	70
Hybrid	1	0			1	0	0	0	0	0	14	0		0	0	18	2	1	0	1	0	9	4	0	0	0	5	13
Complex	72	100			3	10	100	17	3	10	11	0		1	1	54	4	1	0	99	0	4	9	97	69	8	3	13
Unoccupied	3	0			5	88	0	30	2	0	0	0		0	5	0	0	0	0	0	0	0	0	0	24	92	92	4
Fucosylation	36	100			3	10	0	17	3	10	8	0		1	1	63	4	1	0	71	0	3	10	97	68	8	1	14
Sialylation	26	25			2	5	0	1	1	5	12	0		0	0	40	0	0	0	56	0	5	4	57	53	0	4	6
core	6	0			1	0	0	1	1	1	1	0		0	0	2	0	0	0	0	0	1	0	2	1	0	0	1

Total	
Mannose	49
Hybrid	3
Complex	25
Unoccupied	12
Fucosylation	19
Sialylation	11
core	1

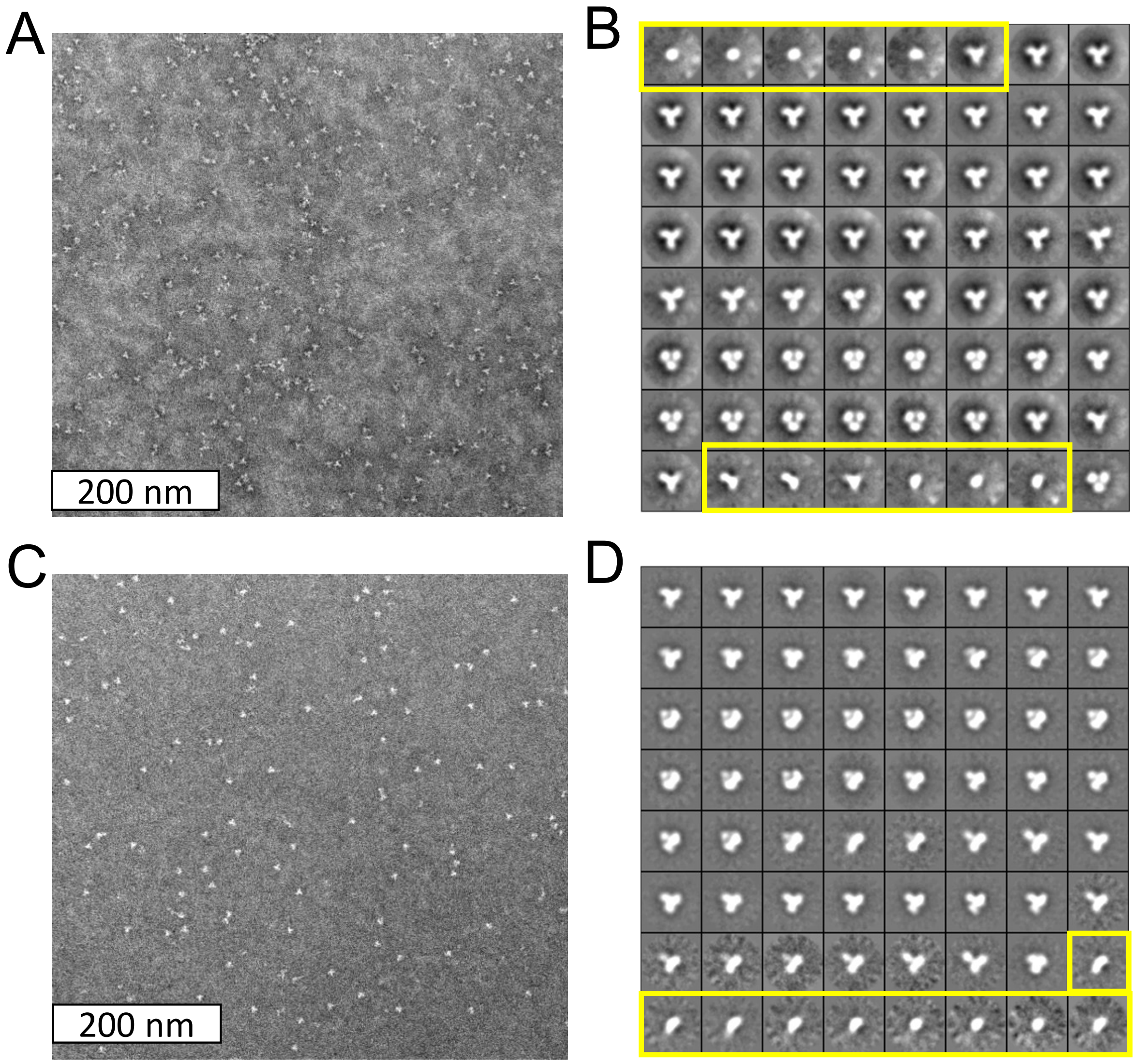

Supplementary Figure 1: Representative of NS-EM image of BG505.664 Env particles secreted by HEK293F cells transfected by plasmid DNA. A) NS-EM image of Env purified by GNL beads. B) 2D class averages of the particles shown in A. Amongst 8071 particles imaged, 20% of these particles fall with in class averages corresponding to non-native conformations shown in yellow. C) NS-EM image of Env purified by GNL followed by size-exclusion chromatography. D) 2D class averages of the particles shown in C. Amongst 6035 particles imaged, 14% of these particles fall with in the class averages corresponding to non-native conformations shown in yellow.

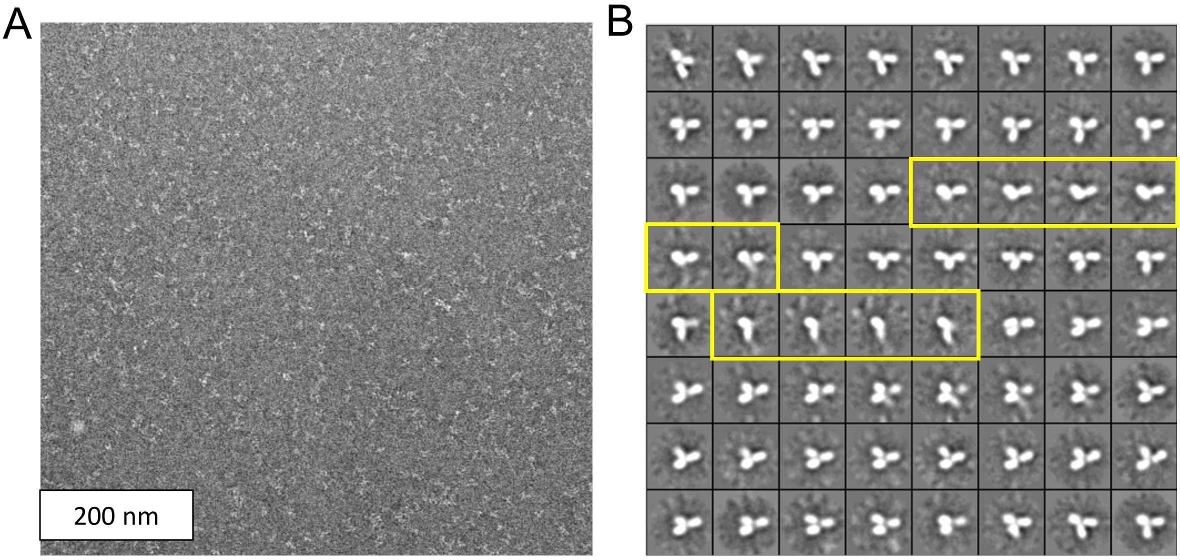

Supplementary Figure 2: Representative NS-EM image of BG505 NFL.664 Env particles secreted by C2C12 cells transfected by replicon RNA. A) NS-EM images of Env purified by VRC01 (CD4 binding site antibody). B) 2D class averages of the particles. Amongst 10679 particles imaged, 17% of these particles fall with in the class averages corresponding to the non-native conformation of Env (highlighted, yellow).

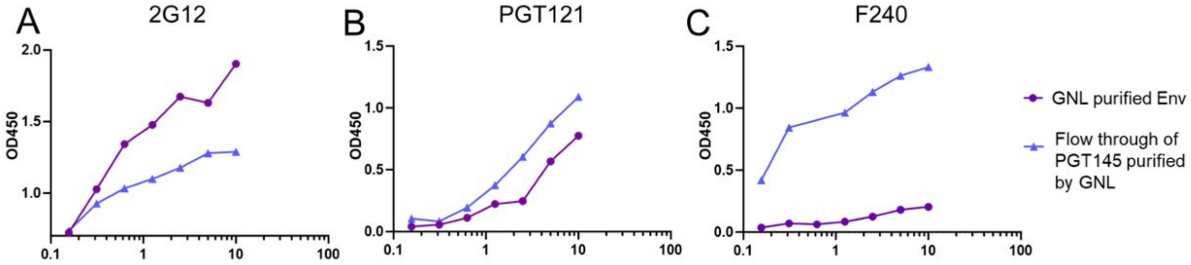

Supplementary Figure 3: Binding analysis of replicon expressed Env affinity-purified using GNL and PGT145. The binding analysis in purple represents the Env purified using GNL beads. The binding analysis in blue represents the non-trimeric conformation of Env which was eluted during PGT145 purification, subsequently purified using GNL. The ELISA was performed with the glycan binding bnAb (A) 2G12, V3 glycan dependent bnAb (B) PGT121, and non-nAb (C) F240. The assays were conducted in duplicate, with each sample being tested twice in independent wells to confirm consistency of results.

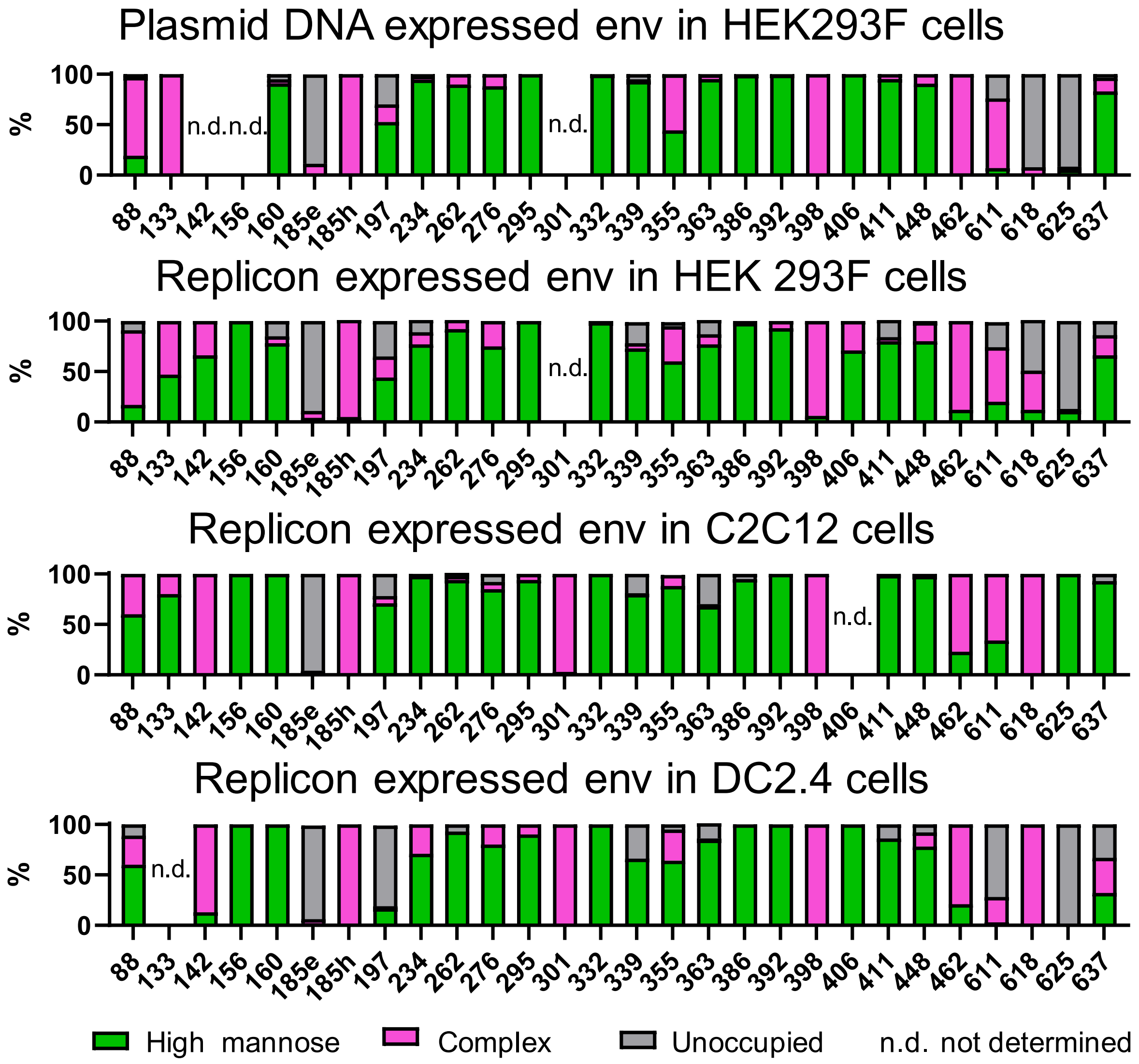

Supplementary Figure 4: Site-specific glycan composition of env via different production systems. The bar graphs represent the glycan composition observed at each N-glycan site with the average of three or more biological replications produced in different production systems. The glycan compositions are represented in three categories, underprocessed glycan structures which includes both oligomannose-type and hybrid-type glycans, complex-type which includes highly processed glycan compositions and core-truncated structures, and unoccupied which represents N-glycan site with no glycan on it. Sites annotated with n.d. represent sites where data was insufficient, and the glycan compositions were consequently not determined.

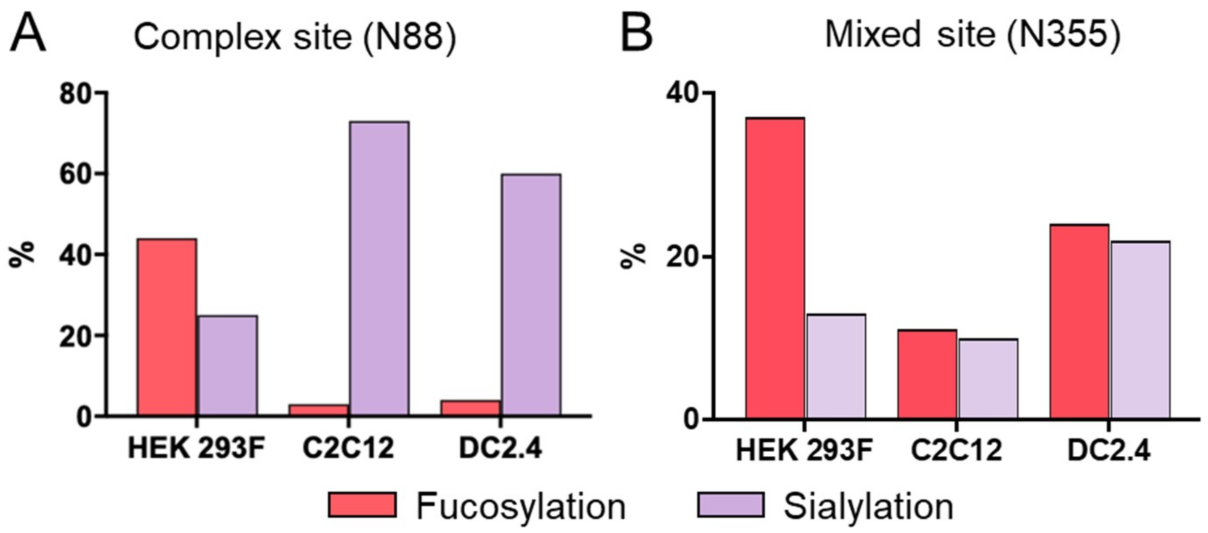

Supplementary Figure 5: Complex-type glycan abundances in replicon expression of env observed across different cell lines. The levels of fucosylation and sialylation observed on env expressed in HEK 293F, C2C12 and DC2.4 cells of the two N-linked glycan sites A) Complex site, N88 and B) Mixed site, N355, are displayed. The bar graphs represent the percentage of fucosylation and sialylation represented in pink and purple, respectively.

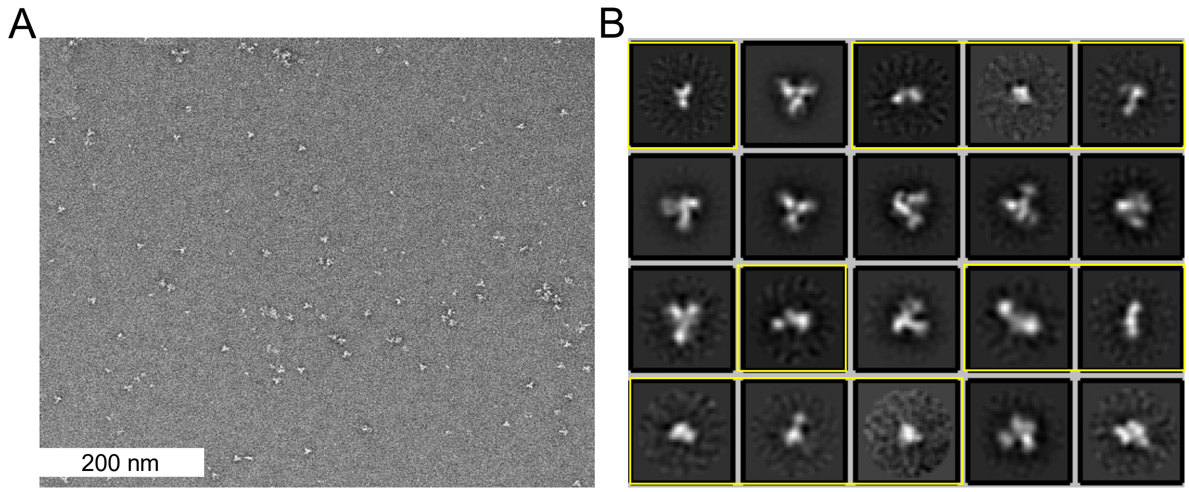

Supplementary Figure 6: A) Representative of NS-EM image of BG505 NFL.664 mutant Env particles secreted by HEK 293F cells transfected with replicon RNA. **B)** 2D class averages of the particles. Amongst all of the particles imaged, 55% fall within class averages corresponding to non-native conformations shown in yellow.

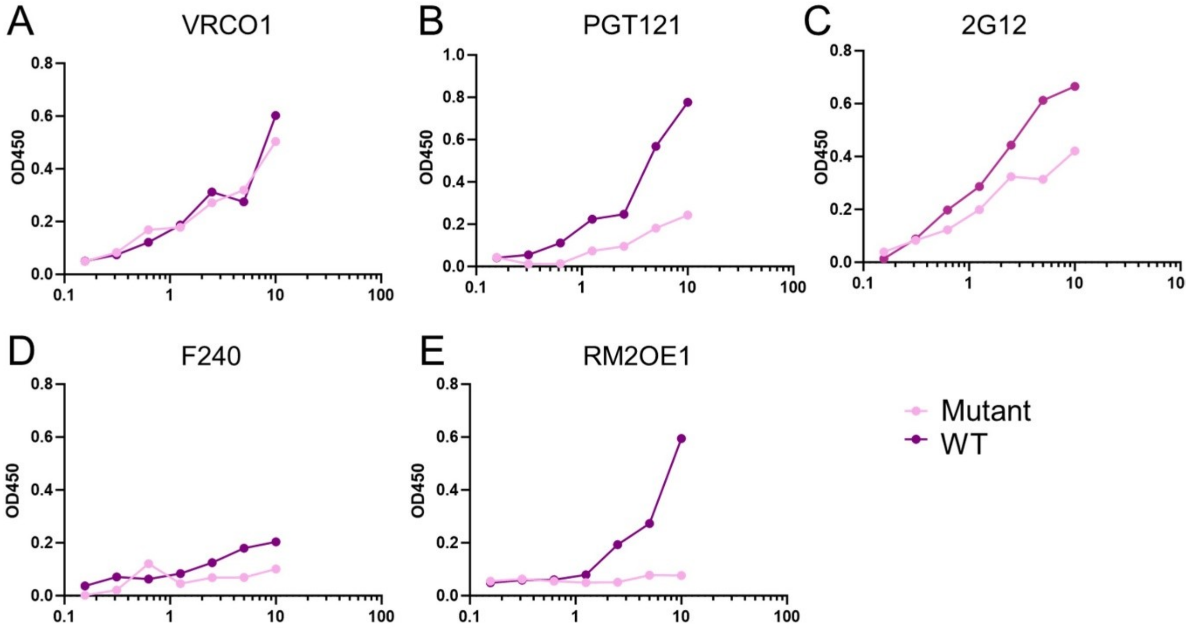

Supplementary Figure 7: Binding analysis of replicon expressed WT and Mutant BG505 NFL.664 with HIV-1 bnAbs and non-nAbs. The bnAbs shown in this figure are VRC01 (A), PGT121 (B), and 2G12 (C). The non-nAbs shown here are F240 (D) and RM20E1 (E). The assay was performed twice to confirm consistency of the results.
